# A 40-Year-Old Woman with Asymmetric Arthritis and Skin Lesions

**DOI:** 10.31138/mjr.30.3.190

**Published:** 2019-09-30

**Authors:** Konstantina A. Bounia, Georgia M. Konstantopoulou, Stamatis-Nick C. Liossis

**Affiliations:** 1 Patras University Hospital, Department of Internal Medicine, Division of Rheumatology, Rion, Patras, Greece,; 2 University of Patras Medical School, Rion, Patras, Greece

**Keywords:** arthritis, pyoderma gangrenosum, Crohn’s Disease

## Abstract

A 40-year old woman with recent asymmetric arthritis and fever was evaluated in our clinic. NSAIDs were recommended, but a few days later she was admitted to our hospital because of worsening arthritis along with the appearance of new skin lesions in both feet. Although she was treated with antibiotics and high dosages of steroids, her arthritis did not improve. The skin lesions progressed from bullous initially to ulcerative pyoderma gangrenosum, so we suggested endoscopic examination of the colon which revealed Crohn’s disease. The patient received I.V. treatment with infliximab resulting in a remarkable response. Some patients with Crohn’s disease may present with extraintestinal manifestations well before the bowel disease is manifested and diagnosed.

## INTRODUCTION

The combination of arthritis and skin lesions represent a usual presentation in our daily clinical practice. Fever can also be an accompanying symptom. In such cases, differential diagnosis includes connective tissue diseases,^2^ infections, endocrine,^3^ hematologic^4^ and malignant diseases^5^

## CASE PRESENTATION

A 40-year-old woman was evaluated in our rheumatologic clinic because of joint pain in the lower extremities during the last week. She mentioned that 3 months ago, she had visited the emergency department of our hospital because of joint pain in the right heel and ankle. She was prescribed NSAIDs with total remission of her symptoms.

**Figure 1.**
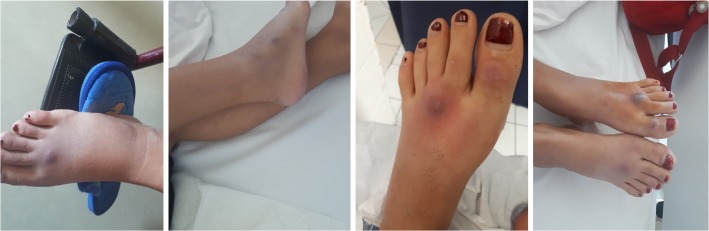
Painful nodules of the feet and right ankle.

On physical examination synovitis of ankles, right knee, right 1^st^ metatarsophalangeal joint and right elbow was seen. She was prescribed naproxen for 2 weeks. One week later she was admitted to the hospital because of persisting arthritis of the legs and feet and Achilles tendonitis of both feet. In addition, purple, bullous and painful lesions at the dorsal surface of both feet had appeared recently (*Figure 1*).

A laboratory work-up showed an elevated CRP (10 mg/dL, normal < 0.8 mg/dL) and ESR (90 mm/h) and a normochromic, normocytic anaemia (Hb=10.9 g/dL). Testing for autoantibodies, viruses, a vaginal and cervical smear, cultures of blood and skin lesion exudates were all negative, as was a purified protein derivative (PPD) skin test. The patient was thoroughly investigated to rule out infections and malignancies. Computed tomographies of chest and abdomen, as well as the echocardiogram, were normal. Mammography and an ultrasound of breasts revealed incidentally a fibroadenoma of the left breast. Biopsy of the skin lesions could not be performed because of their position and possible damage of the underlying tendons.

Although she was treated with wide spectrum antibiotics (even against gonorrhoea), her arthritis and skin lesions were aggravated.

**Figure 2.**
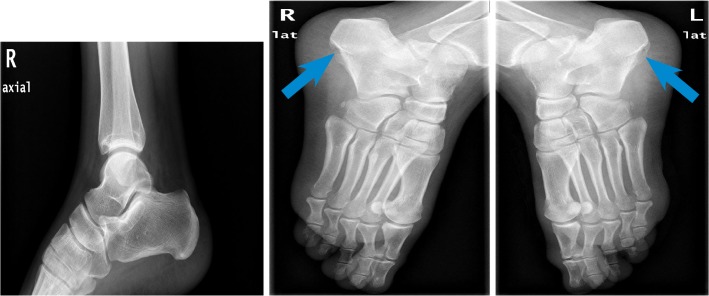
X-rays of the feet during hospital admission showing enthesitis (blue arrows) on both calcanei (film B and C). In contrast, 3 months before she referred to our clinic there is no sign of enthesis involvement (film A).

We examined the patient and taking into account the asymmetric arthritis, the pyoderma gangrenosum lesions of the feet and the radiological image of enthesitis of both heel bones (*Figure 2*), our working diagnosis was a seronegative spondylarthritis. Therefore, we suggested she should undergo a colonoscopy, even though the patient declined having bowel symptoms. Meanwhile, we increased the steroids dosage she was receiving from 16 mg of methylprednisolone p.o. daily to 40 mg of methylprednisolone IV.

**Figure 3.**
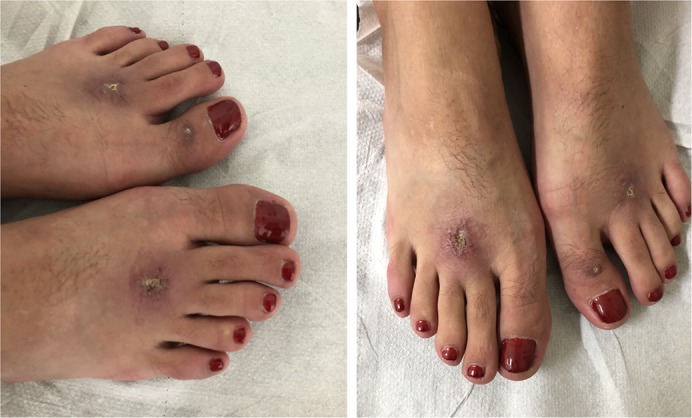
Skin lesions greatly improved after two infusions of Infliximab.

**Figure 4.**
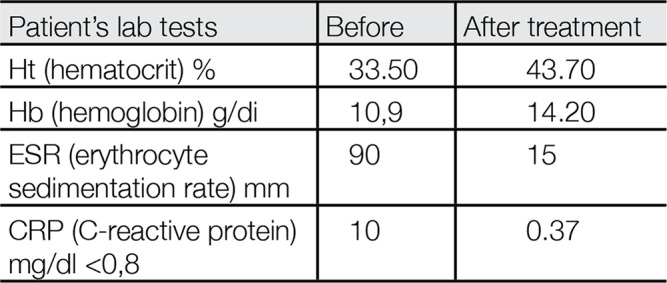
Great improvement of complete blood count and acute phase reactants of the patient a couple of months after treatment with infliximab.

Colonoscopy was performed and established the diagnosis of florid Crohn’s Disease. Treatment with anti-TNF-α agent infliximab (5mg/kg) was initiated and 1 month after the first infusion her arthritis and pyoderma gangrenosum lesions were significantly improved as were the patient’s laboratory tests after a couple of months. (*Figure 3* and *Figure 4*).

## DISCUSSION

We describe herein a patient with Crohn’s disease (CD) with preceding arthritis and pyoderma gangrenosum, without any intestinal manifestations. CD is an inflammatory bowel disease (IBD), which includes extraintestinal manifestations (EIM)^6^ that may or may not be of parallel clinical activity with the bowel disease.^7^ These EIM include manifestations from the musculoskeletal system,^8,9^ skin lesions,^10,11^ aphthous ulcers and eye involvement (uveitis).^8^ The arthritis accompanying CD is usually an asymmetric oligoarthritis affecting mainly large joints with preferential order to knees, ankles, wrists and shoulders. In addition, a symmetric / asymmetric polyarthritis can be presented, affecting small joints independently of IBD presentation and activity.^6,8,13^

Skin lesions of CD include erythema nodosum and pyoderma gangrenosum (PG).^6,14^ It appears that PG has a tendency to appear in females (up to 30% of female patients) and mostly in the lower limbs. It is usually ulcerative when in context of seronegative spondyloarthropathies,^9^ but other types have also been observed, such as nodular, pustular, bullous, or vegetative^10^ in other diseases, too. It has been reported that one quarter of patients with IBD may present with extraintestinal manifestations preceding even 5 months-on average-prior to IBD diagnosis.^6,7^ However, it was surprisingly noted that peripheral arthritis along with PG in particular, do not precede IBD diagnosis as this occurred in our patient.^7^ Anti-TNF-α blockers have been approved for CD management.^17,18^ Furthermore, studies have shown remarkable results in treating the EIM; TNF-α blockers are equally satisfactory in both the musculoskeletal manifestations (peripheral arthritis, axial arthropathy) and skin lesions (erythema nodosum and PG). Our patient had an IBD preceded by an asymmetric arthritis resistant to common/usual therapeutic manipulations (including high doses of steroids) and PG lesions manifested as an unusual (bullous) form that eventually progressed to the ulcerative type. Anti-TNF-α therapy improved the patient’s disease remarkably and quickly, immediately following the first i.v. infusion.

In conclusion, the diagnostic approach and management of patients with arthritis and skin lesions can be challenging. A detailed history, a thorough clinical examination and relevant problem-oriented tests and a careful follow-up can help us establish the proper diagnosis and treatment.
